# Sparing the contralateral submandibular gland without compromising PTV coverage by using volumetric modulated arc therapy

**DOI:** 10.1186/1748-717X-6-74

**Published:** 2011-06-16

**Authors:** Patricia Doornaert, Wilko FAR Verbakel, Derek HF Rietveld, Ben J Slotman, Suresh Senan

**Affiliations:** 1 Department of Radiation Oncology, VU University Medical Center, PB 7057, 1007 MB Amsterdam, The Netherlands

**Keywords:** submandibular gland sparing, volumetric modulated arc therapy, RapidArc, head and neck cancer, dose distribution, xerostomia

## Abstract

**Background:**

Salivary gland function decreases after radiation doses of 39 Gy or higher. Currently, submandibular glands are not routinely spared. We implemented a technique for sparing contralateral submandibular glands (CLSM) during contralateral elective neck irradiation without compromising PTV coverage.

**Methods:**

Volumetric modulated arc therapy (RapidArc™) plans were applied in 31 patients with stage II-IV HNC without contralateral neck metastases, all of whom received elective treatment to contralateral nodal levels II-IV. Group 1 consisted of 21 patients undergoing concurrent chemo-radiotherapy, with elective nodal doses of 57.75 Gy (PTV_elect_) and 70 Gy to tumor and pathological nodes (PTV_boost_) in 7 weeks. Group 2 consisted of 10 patients treated with radiotherapy to 54.45 Gy to PTV_elect _and 70 Gy to PTV_boost _in 6 weeks. All clinical plans spared the CLSM using individually adapted constraints. For each patient, a second plan was retrospectively generated without CLSM constraints ('non-sparing plan').

**Results:**

PTV coverage was similar for both plans, with 98.7% of PTV_elect _and 99.2% of PTV_boost _receiving ≥95% of the prescription dose. The mean CLSM dose in group 1 was 33.2 Gy for clinical plans, versus 50.6 Gy in 'non-sparing plans' (p < 0.001). In group 2, mean CLSM dose was 34.4 Gy for clinical plans, and 46.8 Gy for non-sparing plans (p = 0.002).

**Conclusions:**

Elective radiotherapy to contralateral nodal levels II-IV using RapidArc consistently limited CLSM doses well below 39 Gy, without compromising PTV-coverage. Future studies will reveal if this extent of dose reduction can reduce patient symptoms.

## Background

Bilateral nodal irradiation is indicated in patients with head and neck cancer who present with either locally advanced disease, or a tumor located in the midline. Irradiation delivered using conventional, non-intensity modulated techniques in these patients generally leads to a high degree of xerostomia [1]. Xerostomia is a major cause of morbidity following radiotherapy in patients with head and neck cancer, and arises due to irradiation of both major and minor salivary glands [2]. It causes physical difficulties in swallowing and speaking, altered taste, predisposes to early dental caries, and is considered by patients to be a major cause of reduced quality of life (QoL) [3].

The use of intensity-modulated radiotherapy (IMRT) has allowed for reduction of doses to the parotid glands (PG) without compromising tumor coverage, and many authors have reported a reduction in xerostomia [4-14]. Although nearly two thirds of the stimulated saliva is produced by the PG, submandibular (SM) glands are largely responsible for salivary output in unstimulated conditions [15]. Unlike the PG which produces mainly serous secretions, SM glands produce mixed serous and mucinous secretions, and the latter accounts for a patient's subjective sense of moisture. Approaches that reduce doses to at least one SM gland can reduce the incidence of xerostomia [16,17]. Furthermore, there seems to be a better correlation between the incidence of xerostomia and the mean dose to the PGs and SM glands taken together as one organ, than to the PGs alone [18].

The drawbacks of conventional IMRT delivery are well recognized, with longer delivery times decreasing patient throughput [19], and an increase in the volume of normal tissues receiving low doses of radiation. Volumetric modulated arc therapy called RapidArc™ was introduced into clinical care in 2008 [20-23], and it uses continuous changes in the dose rate, the shape of the beam, and speed of gantry rotation to permit faster delivery of highly modulated IMRT plans [24]. Consequently, we implemented treatment plans that specifically spared both the PG and the contralateral submandibular gland (CLSM) in patients without contralateral (CL) neck metastases requiring bilateral neck irradiation. The present report describes the planning and clinical characteristics of 31 HNC patients treated in this manner, all of whom also had comparative plans without CLSM.

## Methods

### Patient selection

Use of RapidArc at our department commenced in 2008, and we developed a constraint set aiming to spare the CLSM gland in 2009. The present study reports on the first 31 patients who were treated with clinical sparing of the CLSM gland. All patients had stage II-IV HNC without CL lymph node metastases (except for 1 patient who had one level IV positive node) and received elective treatment to CL nodal levels II-IV (Table 1).

**Table 1:** Patient characteristics

Patient	Site	subsite	TNM	stage	elective dose (Gy)	chemotherapy
1	oropharynx	tonsil	T4N2b	IV	57.75	TPF + carbo weekly
2	oropharynx	base of tongue	T3N0	III	57.75	CDDP 3x
3	oropharynx	tonsil	T3N0	III	57.75	cetuximab
4	oropharynx	tonsil	T2N2b	IV	57.75	CDDP weekly
5	oropharynx	tonsil	T4bN0	IV	57.75	TPF + CDDP weekly
6	oropharynx	base of tongue	T3N2b	IV	57.75	CDDP 3x
7	oropharynx	tonsil	T3N1	III	57.75	CDDP 3x
8	oropharynx	tonsil	T2N2b	IV	57.75	CDDP 3x
9	oropharynx	pharyngeal wall	T3N2b	IV	57.75	CDDP 3x
10	oropharynx	tonsil	T3N2b	IV	57.75	CDDP 3x
11	oropharynx	tonsil	T2N2b	IV	57.75	CDDP 3x
12	oropharynx	base of tongue	T2N1	III	57.75	CDDP 3x
13	oropharynx	tonsil	T4N1	IV	57.75	TPF + carbo weekly
14	oropharynx	base of tongue	T3N0	III	57.75	cetuximab
15	larynx	supraglottis	T3N1	III	57.75	CDDP weekly
16	larynx	supraglottis	T3N0	III	57.75	CDDP 3x
17	larynx	supraglottis	T4N0	IV	57.75	CDDP 3x
18	larynx	supraglottis	T3N0	III	57.75	CDDP 3x
19	hypopharynx	postcricoid	T3N2c	IV	57.75	CDDP 3x
20	hypopharynx	piriform sinus	T4aN2b	IV	57.75	CDDP 3x
21	hypopharynx	piriform sinus	T4N2b	IV	57.75	CDDP 3x
22	oropharynx	tonsil	T2N0	II	54.25	
23	oropharynx	base of tongue	T1N1	III	54.25	
24	oropharynx	tonsil	T2N0	II	54.25	
25	oropharynx	base of tongue	T3N0	III	54.25	
26	oropharynx	soft palate	T2N1	III	54.25	
27	oropharynx	soft palate	T1N0	I	54.25	
28	larynx	transglottis	T2N2b	IV	54.25	
29	larynx	glottis	T3N0	III	54.25	
30	larynx	supraglottis	T2N0	II	54.25	
31	hypofarynx	piriform sinus	T2N1	III	54.25	

CDDP: cisplatinum

CDDP 3x: 3 cycles of cisplatinum 100 mg/m^2^

TPF: docetaxel 75 mg/m^2 ^day 1, cisplatinum 75 mg/m^2^day 1, 5-FU 750 mg/m^2 ^day 1-5

Group 1 consisted of 21 patients who underwent concurrent chemoradiotherapy, including 14 patients with oropharyngeal cancer, 4 larynx cancer and 3 hypopharynx cancer. The majority (n = 14) received three cycles of concurrent single-agent cisplatin 100 mg/m2. Three patients received induction chemotherapy (taxotere- cisplatin -5-FU) followed by weekly cis- or carboplatin, 2 patients received cisplatinum 40 mg/m2 weekly, and 2 patients were only fit to receive concurrent radiotherapy with cetuximab. Group 2 consisted of 10 patients who were treated using only accelerated radiotherapy (6 fractions/week), and included oropharynx (n = 6), larynx (n = 3) and hypopharynx (n = 1) cancer.

All patients were positioned in a 5 point fixation mask (Posicast^® ^Thermoplastics, Civco Medical Solutions). The gross tumor volume (GTV) was delineated on a contrast-enhanced planning CT scan acquired with 2.5-mm slice thickness. Target volumes were defined by co-registration of diagnostic MRI scans and/or PET scans. The gross tumor volume (GTV) was defined as the primary tumor and involved lymph nodes on imaging and examination under anesthesia. The 'boost' clinical target volume (CTV_boost_) comprised the GTV with a margin of 0.5 cm, and was corrected for anatomical boundaries. The 'elective' CTV (CTV_elect_) included the CTV_boost _plus 0.5 cm and bilateral elective lymph nodes: CL levels II-IV and at least IL levels II-V, and level I, VI and/or retropharyngeal nodes in accordance with published guidelines [25,26]. A margin of an additional 3 (upper part) to 5 (shoulder region) mm was taken to create planning target volumes (PTVs).

### Planning objectives and techniques

The objectives used for the target volumes and organs at risk are summarized in Table 2. In group 1, dose prescription was set to 57.75 Gy at 1.65 Gy/fraction to the PTV_elect _and 70 Gy at 2 Gy/fraction to the PTV_boost _delivered as a simultaneous integrated boost (SIB). In group 2, patients received 54.25 Gy at 1.55 Gy/fraction tot the PTV_elect_. A standard constraint set was used for RA optimization, aiming to achieve at least. 95% of the boost dose in 99% of the PTV_boost _and 95% of the elective dose in 98% of the PTV_elect_, while keeping the boost and elective volumes receiving >107% of the prescribed dose as small as possible. This constraint set is similar to that described previously [24], except for the addition of objectives to spare the CLSM gland. The maximum dose specified for the spinal canal was 36-40 Gy. Priorities for the PTVs, spinal cord and salivary glands were 120-130, 125 and 80 respectively. Four dose objectives were set for both PGs and the CLSM gland, and only those objectives were interactively adapted for each individual patient during the first 2 to 3 levels of a 5-level multi-resolution optimization process that aimed to keep the mean CLSM dose low without compromising PTV coverage. The built-in normal tissue objective with a priority of 80, and 3 constraints on a 1 cm thick ring created around the PTVs were used to enforce a steep dose fall-off outside the target volumes.

**Table 2:** Planning objectives/constraint set

Target Volume	min dose (Gy)	max dose (Gy)	priority
PTV elective (1.65 Gy/fr)	57	58.5	120-130
PTV elective (1.55 Gy/fr)	53.5	55	120-130
PTV boost	69	71	120-130
spinal cord		36	125
standard ring	DVH		90-110
parotids	DVH, adapt during first iterations		80
CLSM	DVH, adapt during first iterations		80
Shoulders		20	75

Optimization and dose calculations were performed using the Eclipse treatment planning system (version 8.2.23) in 10 patients, and subsequently Eclipse version 8.6.15 for 21 patients (Varian Medical Systems, Palo Alto). Treatment delivery was performed using 6 MV photon beams from a Varian 2300 linac with the Millennium 120-MLC. The Anisotropic Analytical Algorithm (AAA) photon dose calculation algorithm was used with a calculation grid of 2.5 mm. Each plan consisted of 2 coplanar arcs of 358° (one counterclockwise (CCW), one clockwise (CW)). In the first 10 patients, a sequential approach was used, in which the first arc plan was used as a base dose plan for the second arc plan which compensated for possible under- or overdosage in the first arc plan, leading to a homogeneous dose in the PTV [23]. In the last 21 patients, the 2 arcs were simultaneously optimized. To appreciate the target coverage in the areas where the PTV approaches the surface, a local build-up of 6 mm (to overcome dose build-up under the skin) was used for optimization purposes.

For plan evaluation, the boost and elective volumes receiving at least 95% of the prescribed doses (V95), as well as the V107, were registered. This was done in the plans using the local build-up for optimisation purposes. A conformity index (CI), which was defined as the ratio between the volume receiving at least 95% of the prescribed boost dose and the volume of the PTV_boost_, was calculated. The mean doses of both PGs, the CLSM gland and the maximum dose to the spinal canal were also registered.

In order to confirm the results achieved in our initial 31 patients, the CLSM doses of 25 consecutive patients treated subsequently using the same technique, were also analyzed. All patients had stage II-IV disease, were treated electively to the CL levels II-IV and received a dose of 70 Gy to the PTV_boost _and 54.25 Gy to the PTV_elect_.

### Planning study

For purposes of the present analysis, a second plan was retrospectively generated using identical constraints on all volumes except the CLSM gland (referred to as the 'non-sparing plan'). Doses to the PTVs, PGs, CLSM gland and spinal canal were registered and compared for both plans. Volumes encompassed by the 95% isodose line (V95) of the elective and boost dose were generated for the sparing and non-sparing plans and compared by using the Wilcoxon signed-ranks test. P < 0.05 was considered as significant.

### Quality assurance (QA)

Individual plan QA was performed for all patients. For 10 patients, dose distributions were measured using Gafchromic^® ^EBT films inserted in 1-3 coronal planes of a cube polystyrene phantom, allowing dose verification during a single treatment session [23]. In the remaining 21 patients, QA was performed using the MatriXx ionization chamber array (IBA, Louvain-la-Neuve, Belgium) in one coronal plane of an in-house designed polystyrene phantom. The coronal planes were selected to measure a combination of boost and elective doses. All measurements were performed for the combination of the two arcs and they were compared to the calculated dose of the same patient plan on the CT-scan with the respective phantom. A gamma-evaluation was performed, using dose differences of 3% and distance to agreement of 2 mm.

Routine patient set-up was performed using two orthogonal kV-images (OBI, Varian Medical Systems) performed prior to each of the first 3 fractions, which were registered to digitally computed radiographs from the planning CT-scan using translations only. After the fourth fraction, patients were positioned according to the mean of the first 3 set-ups. The set-up procedure was repeated after 20 fractions, and a cone beam CTscan (CBCT) was then performed to ensure PTV coverage.

### Toxicity and quality of life assessment

Patients are all included in a standardized follow-up program with weekly evaluation by the radiation oncologist and scoring of toxicity according to the RTOG Radiation Morbidity Scoring Criteria [27]. Health-related QoL was routinely assessed using the EORTC QLQ-C30 and H&N35 questionnaires at baseline, 1 and 6 months post treatment and at 6-month intervals thereafter [28,29].

## Results

All 31 patients completed CLSM sparing radiotherapy as was planned. Median follow up was 19 months (range 14-25 months). To date, no regional recurrences have been observed. Two patients developed a local recurrence and underwent a total laryngectomy. Two patients had a local recurrence with lung metastases. One patient developed lung metastases only.

### Target coverage

The mean volumes of PTV_elect _and PTV_boost _(588 cm^3 ^and 178 cm^3^, respectively) for patients in group 1 were similar to that in group 2 (565 cm^3 ^and 118 cm^3^). Mean PTV coverage, CI and OAR doses for both the sparing and the non-sparing plans, are summarised in Table 3. The PTV coverage of all 31 patients studied was excellent, with on average 98.7% of PTV_elect _and 99.2% of PTV_boost _receiving ≥ 95% of the prescription dose. In 'non-sparing' plans, the corresponding PTV coverage was 98.9% and 99.2%, respectively. For both plans, on average, only 0.8% of PTV_boost _received >107%. The resulting plan CI was 1.18 in group 1, and 1.14 in group 2 (not different from the CI 'non-sparing' plans).

**Table 3:** Results

Site		SM CL	SM CL (non-sparing)	PG IL	PG IL (non-sparing)	PG CL	PG CL (non-sparing)	Sp C	Sp C (non-sparing)	V B ≥95%	V E ≥95%
A	mean	33.7	51.5	34.2	33.3	21.8	21.4	42.1	41.9	99.3	98.9
	range	(46.5-57.3)	(24.7-41.6)	(16.6-54.3)	(16.3-53.2)	(12.2-31.8)	14.2-32.1)	(37-50)	(35.8-49)	(98.9-99.9)	(98.2-99.5)
B	mean	32.4	49	27.9	27.8	22	21.9	36.4	36.3	99.7	98.5
	range	(44.3-52.8)	(29.7-39.5)	(16.4-55.5)	(16-55.7)	(16.7-30.6)	(16.7-30.3)	(23-49.8)	(23-49.8)	(99-99.9)	(97.9-98.9)
C	mean	35.1	47.1	28.7	29.1	21.9	22	40	39.8	98.5*	98.9
	range	(43.7-52.4)	(26.6-40.6)	(16.5-48.2)	(17-46.3)	(13.6-30.9)	(14.5-30.7)	(30-44.9)	(29.7-45.3)	(96.2-99.6)	(97.9-98.9)
D	mean	33.4	46.4	21.2	21	16.2	16	36.4	36	99.6	98.3
	range	(42.2-51.8)	(30-36.8)	(12.7-35.7)	(12.4-35.6)	(10.7-26.1)	(10.3-26.3)	(24.7-41)	(24.4-41.2)	(99.1-99.9)	(97.6-98.9)

A: oropharynx, elective dose 57.75 Gy (14 patients) Doses in Gy

B: larynx/hypopharynx, elective dose 57.75 Gy (7 patients) Sp C: spinal cord

C: oropharynx, elective dose 54.25 Gy (6 patients)

D: larynx/hypopharynx, elective dose 54.25 Gy (4 patients)

V B ≥95%:% of PTV boost receiving ≥95% of the prescribed dose

V E ≥95%:% of PTV elective receiving ≥95% of the prescribed dose

*: in 1 patient with T1 carcinoma of the soft palate, 96.2% of PTV_boost _received 95% of prescribed dose, this was accepted because of the fact that a large part of the PTV_boost _consisted of air.

**Figure 1:**
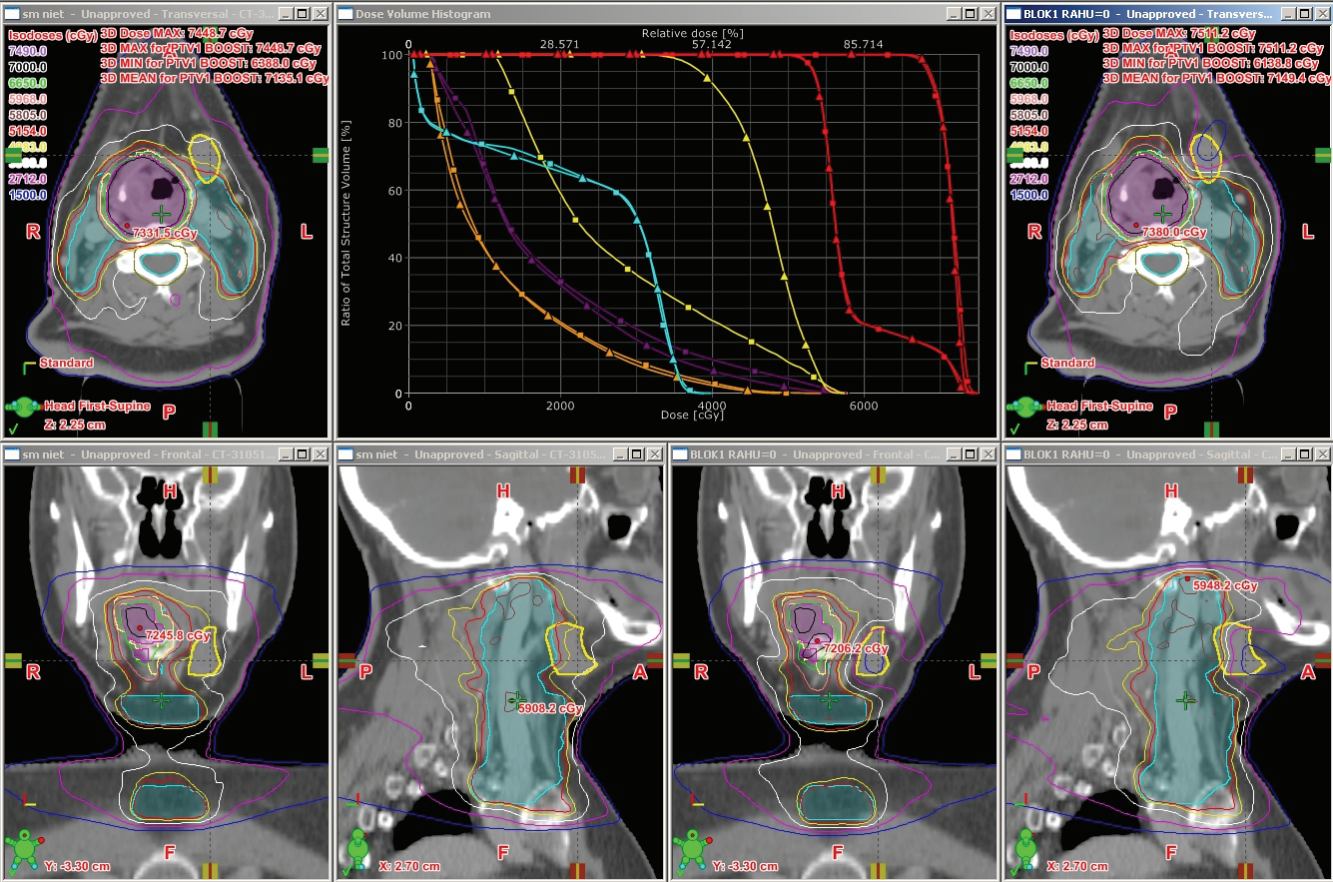
**Dose distributions and DVH for a typical patient with an oropharyngeal tumor**. Comparison of clinical plan (pictures right) with 'non-sparing' plan (pictures left). PTV in magenta, PTV in blue. DVH (■ = clinical plan, ▲ = 'non-sparing' plan) of CLSM in yellow, both PTVs in red, left PG in purple, right PG in orange, spinal cord in blue.

Sparing of the CLSM gland did not significantly reduce the volume encompassed by the 95% isodoseline of the elective or boost doses compared to the non-sparing plans (Wilcoxon signed-ranks test p = 0.16 and p = 0.64 respectively). An example of the clinical and non-sparing plan in a typical patient can be appreciated in Figure 1.

### Organs at risk (OAR)

With the exception of the mean dose in the CLSM gland, no differences in doses to other OARs were observed for clinical and non-sparing plans. In group 1, the mean CLSM gland dose was 33.2 Gy (clinical plans) and 50.6 Gy (non-sparing) (p < 0.001). The maximum dose to the spinal canal was on average 40.2 Gy (compared to 40 Gy in the non-sparing plans). IL and CL parotids received 32.1 Gy and 21.9 Gy respectively (versus 31.5 Gy and 21.5 Gy in the non-sparing plans).

In group 2, the mean CLSM gland dose was 34.4 Gy for clinical plans, and 46.8 Gy for non-sparing plans (p 0.002). The maximum dose to the spinal canal was 38.6 Gy (as opposed to 38.3 Gy in the non-sparing plans). Clinical IL and CL parotid doses were 25.7 Gy and 19.6 Gy respectively, versus 25.8 Gy and 19.6 Gy in non-sparing plans.

In the follow up cohort of 25 patients treated using the same technique, the mean CLSM dose was 32.8 Gy. Both PTV_elect _and PTV_boost _coverage was again excellent, with 98.4% and 99.3% of volumes receiving 95% of the prescription dose.

### Acute toxicity

Acute toxicity observed was consistent with the toxicity seen in patients treated with conventional IMRT delivery to these doses [6]. In group 1 (21 patients), 5 patients experienced RTOG grade 3 cutaneous toxicity (moist desquamation). Half (10) of all patients had a confluent mucositis (RTOG grade 3 toxicity) and 19 patients required opioid analgesia. Fifteen patients developed grade 2 xerostomia with markedly altered taste. Prophylactic placement of a percutaneous endoscopic gastrostomy tube (PEG-tube) was performed in 19 patients, and all but one patient actually used the PEG-tube.

In group 2 (10 patients), 7 patients developed confluent mucositis, and 9 patients experienced a markedly altered taste our dry mouth (xerostomia grade 2). No preventive PEG tubes were placed. Only one patient needed a nasogastric tube. Eight patients required opioids.

### Quality assurance

Film and MatriXx measurements showed that on average only 1.3% of the measurement points exceeded a combination of dose difference > 3% or distance to agreement > 2 mm (range 0-4.6%). For only 6 of the patients, more than 3% of the measured points exceeded this limit.

## Discussion

The pathogenesis of xerostomia is complex and appears to depend on not only PG function, as a discrepancy was noted between preserved PG function measured using objective tools, and subjective patient-reported xerostomia [30-33]. As particularly the mucinous secretions of the SM gland contribute to the subjective feeling of oral hydration, we developed and clinically implemented a technique to spare both the PGs and CLSM gland in patients undergoing elective irradiation to clinically negative CL level II-IV nodes. Our main findings are that reductions in mean dose to the CLSM gland to 33.2 Gy and 34.4 Gy, respectively, is possible in who need to undergo elective doses of 57.75 Gy and 54.25 Gy.

SM glands are located adjacent to the jugulodigastric nodes, which are the first echelon for most HNC tumors. Consequently, SM sparing is infrequently considered for fear of reducing PTV coverage [2]. We observed no compromise in PTV coverage in most patients, although 5 clinical plans had a minor underdosage (to 90% of the prescribed elective dose) in 0.5 cm^3 ^to the PTV_elect _in the vicinity of the CLSM. In all these 5 patients, the coverage of the PTV_elect _met our clinical acceptance criteria as 97.9% - 98.6% was covered by 95% of the elective dose. For the purposes of the current study, plans for all these 5 patients were repeated using a PTV_elect _margin of 5 mm (3 mm standard + 2 mm extra) for the PTV regions directly adjacent to the CLSM gland. In all patients, the volume of underdosage could be reduced to 0-0.2 cm^3^. In 2 patients, the mean CLSM dose remained the same, in 2 patients there was an increase of 1.6 Gy and in 1 patient an increase from 31.8 to 35.7 Gy. Consequently, we currently enlarge the PTV_elect _adjacent to CLSM with an extra 2 mm in order to reduce the likelihood of creating a small rim of underdosage in the PTV_elect_. It is reassuring to note the application of our technique in an additional 25 patients revealed that the reduction in CLSM doses was maintained.

With a short median follow up in our 31 patients of 19 months, no contralateral regional recurrences were observed. Recent work in 285 patients indicates that isolated regional relapse in the elective contralateral neck are very uncommon after the use of IMRT [34]. These authors delivered a dose of 56 Gy in 32 fractions to elective regions, which was comparable to the doses used in our patients. As we found no significant differences between the clinical and the non-sparing plans in PTV receiving at least 95% of the elective and boost doses, the likelihood of recurrences in the vicinity of the CLSM are expected to be low.

Few studies have addressed the dose-response relationships of the SM gland. A study measuring unstimulated whole mouth salivary flow suggested a TD_50 _of 32.6 Gy at 6 months and 34.1 Gy at 12 months for the SM gland [35]. A study in 148 patients treated with IMRT, where no attempt was made to spare the SM glands, resulted in only a limited number of data points in the low-dose region of the SM [36]. Data from this study suggested an exponential mean dose-related decrease in SM output, up to a threshold of 39 Gy, above which little or no recovery of salivary flow was seen.

Other approaches for sparing the SM gland have been reported. A planning study in 10 patients with oropharyngeal cancer which delivered a dose of 54 Gy to the CL PTV_elect_, reported a reduction in mean CLSM gland dose from 54 Gy to 40 Gy [37] However, these authors had to accept an underdosage in the vicinity of the CL PTV_elect _to 90% of the prescribed dose. Surgical transfer of the SM gland into the submental space outside the field can lead to a significant improvement of salivary function [16]. The same authors recently performed a planning study combining SM gland transfer with helical tomotherapy in patients undergoing postoperative RT, and reported achieving a mean dose of 23 Gy in the spared SM gland [38]. In a study where 18 patients actually underwent treatment using plans to limit mean CLSM gland dose to below 26 Gy, no clear definition of the doses in CTVs and PTVs was described [35]. In this study, an underdosage in PTV of up to 10% at the periphery of the CLSM gland was accepted and volumes of underdosage were not reported [39]. Another study recently reported on CLSM sparing in mostly postoperative patients, who comprised 47 of the 52 cases [17]. An impressive reduction in the mean dose to the CLSM gland from 57.4 Gy (non-spared group) to 20.4 Gy (spared group) was described. However, a coverage of 95% of the PTV was considered acceptable and no data on regions of potential underdosage were described. The lower mean CLSM gland dose resulted into a lower RTOG xerostomia score and stimulatated slivary flow rates up to 6 months after therapy (but not thereafter). The authors reported that unstimulated salivary flow rates were significantly better at all time points.

A limitation of our study is the lack of objective assessment of the SM salivary flow, although it must be recognized that the assessment of xerostomia can be subjective and that objective salivary flow measurements may not translate into patient-reported complaints [12,14,30,33]. Since a mean dose of less than 35 Gy to the CLSM gland was achieved, we hope to demonstrate a significant further decrease in patient-scored xerostomia with longer follow-up.

## Conclusion

In HNC patients with a clinically negative contralateral neck, requiring elective RT to the CL nodal levels II-IV, use of RapidArc significantly reduced mean doses to the CLSM gland to below 35 Gy, without compromising PTV coverage. Longer follow-up is required to exclude the potential risk of tumor recurrences in the contralateral neck and to demonstrate a significant decrease in xerostomia.

## Competing interests

The VU University Medical Center has a research collaboration with Varian Medical Systems (Palo Alto, CA).

## Authors' contributions

PD collected the clinical and treatment data, created the "non-sparing" plans, WFARV developed the constraint set, PD and SS drafted the manuscript. All authors contributed to the drafting of the manuscript and approved the final manuscript
